# Supplementary material for: New Bithiophene Extended IDIC-Based Non-Fullerene Acceptors and Organic Photovoltaics Thereof
Source: Molecules. 2022 Feb 7;27(3):1113. doi: 10.3390/molecules27031113 (PMC8838869; doi:10.3390/molecules27031113)
Supplement: Supplementary file 1 [file molecules-27-01113-s001.zip › molecules-1545908-supplementary.pdf]

New Bithiophene Extended IDIC-Based Non-Fullerene Acceptors and Organic Photovoltaics Thereof

Yeong Heon Jeong [1,†], Jae Min Jeon [2,†], Jun Young Kim [2,*] and Yun-Hi Kim [1,*]

[1] Department of Chemistry and RINS, Gyeongsang National University, Jinju 660-701, Korea; dudgjs02@naver.com
[2] Department of Semiconductor Engineering, Gyeongsang National University, Jinju 660-701, Korea; jmjeon95@gnu.ac.kr
* Correspondence: kimjy86@gnu.ac.kr (J.Y.K.); ykim@gnu.ac.kr (Y.-H.K.);
Tel.: +82-55-772-1732 (J.Y.K.); +82-55-772-1491 (Y.-H.K.)
† These authors contributed equally to this work.

Supplementary Material

Experimental Section

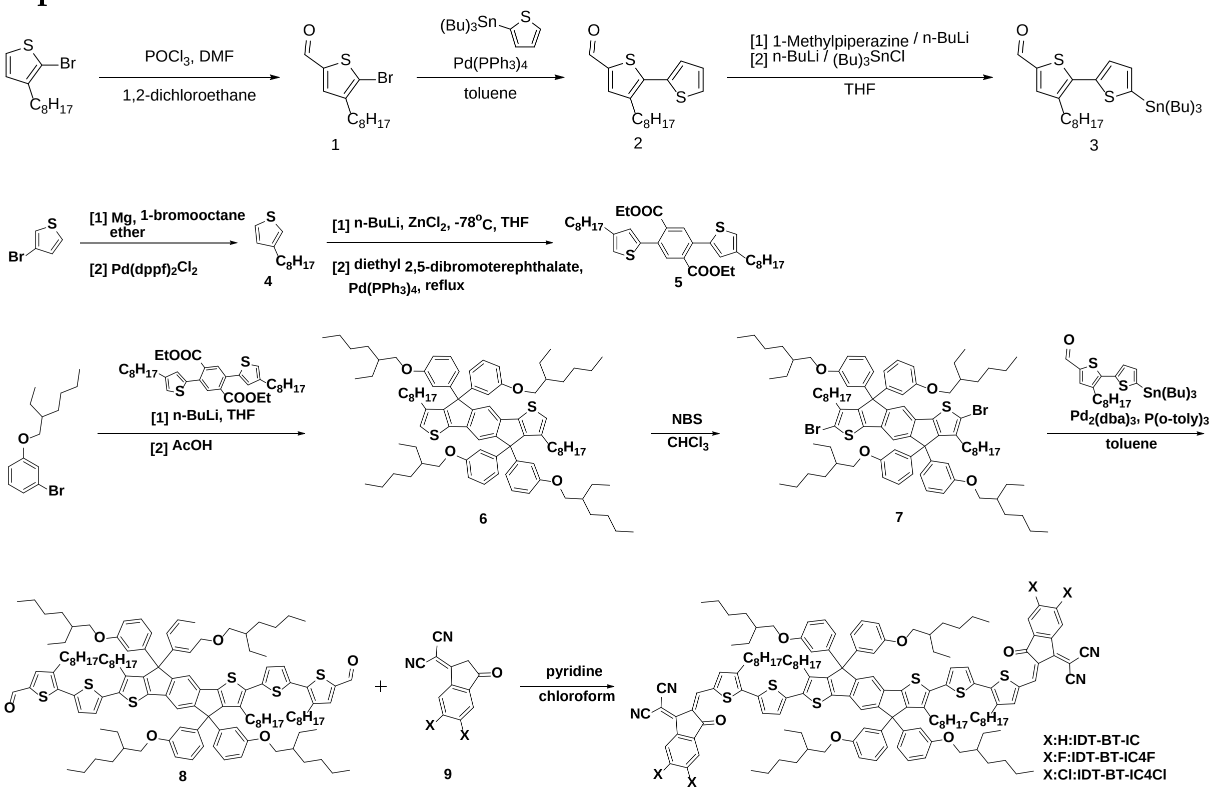

Measurement

^{1}H NMR spectra were acquired using a Bruker 300 FT-NMR spectrometer and ^{13}C NMR spectra were recorded using a Bruker DRX 500 MHz spectrometer. HR mass analysis were obtained by a Joel JMS-700. Thermal gravimetric analysis (TGA) were performed using a TA 2050 TGA thermogravimetric analyzer under a nitrogen atmosphere with a heating rate of 10 °C/min. Differential scanning calorimetry (DSC) was conducted under nitrogen atmosphere using a TA instrument 2100 DSC. The samples were heated at 10 °C/min from 10 °C to 300 °C. UV–vis absorption spectra were measured using a Perkin-Elmer LAMBDA-900 UV/vis/IR spectrophotometer. Cyclic voltammetry (CV) was

performed at a room temperature in a 0.1 M solution of tetrabutylammonium perchlorate (Bu_4NClO_4) in Chloroform under nitrogen gas at a scan rate of 50 mV / s. A Pt wire was used as the counter electrode and an Ag/AgCl electrode as the reference electrode.

Materials

Some starting materials were purchased from TCI. Tris(dibenzylideneacetone)dipalladium (0) was purchased from Umicore. Three kinds of benzothiadiazole monomers **(9),** diethyl 2,5-dibromoterephthalate, 3-bromothiohene, 1-bromooctane and 2-bromo-3-octylthiophene were purchased from Boomking. All reagents purchased commercially were used without further purification. Tetrahydrofuran (THF) and diethyl ether were dried over sodium and benzophenone.

Synthesis of 5-bromo-4-octylthiophene-2-carbaldehyde (1).

DMF (34.48 g, 471.7 mmol) was added to stirring solution of 2-bromo-3-octylthiophene (12.98 g, 47.17 mmol) in dried 1,2-dichloroethane (140 ml) under N_2 atmosphere. After dropping $POCl_3$ (72.32 g, 471.7 mmol) at 0°C, the solution was refluxed for 8h. After cooling to ambient, water was added and the mixture was extracted with dichloromethane, and washed with water, dried with $MgSO_4$ and concentrated. The residues was purified by column chromatography using methylene chloride:n-hexane (v:v=2:1) as eluent to get the product as an yellow oil (10.3 g, 72%). ^{1}H NMR (300 MHz, CD_2Cl_2, δ): 9.77-9.78 (d, *J* = 1 Hz, 1H), 7.52-7.56 (d, *J* = 2 Hz, 1H), 2.61-2.67 (t, 2H), 1.60-1.67 (m, 2H), 1.31-1.37 (m, 10H), 0.90-0.94 (m, 3H).

Synthesis of 3-octyl-[2,2'-bithiophene]-5-carbaldehyde (2).

In 3-neck round bottom flask, to stirring solution of tri-n-butyl(thiophen-2-yl)stannane (9.13 g, 24.47 mmol) and 5-bromo-4-octylthiophene-2-carbaldehyde (4.95 g, 16.31 mmol) in dried toluene (400 ml), $Pd(PPh_3)_4$ (0.85 g, 0.73 mmol) was added after nitrogen bubbling for 30 minutes. The solution was refluxed for 12 h. After cooling to ambient, water was added and the mixture was extracted with dichloromethane and washed with water, dried with $MgSO_4$ and concentrated. The residues was purified by column chromatography using methylene chloride:n-hexane (v:v=1:1) as eluent to get the product as an pale yellow solid (3 g, 60%).

^{1}H NMR (300 MHz, CD_2Cl_2, δ): 9.86 (s, 1H), 7.66 (s, 1H), 7.49-7.51 (m, 1H), 7.34-7.35 (m, 1H), 7.15-7.18 (m, 1H), 2.81-2.86 (t, 2H), 1.66-1.73(m, 10H), 0.9-0.94(m, 3H). ^{13}C NMR (500 MHz, $CDCl_3$, δ): 182.65, 141.27, 140.46, 140.43, 138.92, 134.95, 127.82, 127.59, 127.36, 31.85, 30.34, 29.44, 29.36, 29.26, 29.22, 22.67, 14.12. HR-MS (EI) m/z $C_{17}H_{22}OS_2$ Calcd : 306.1112, Found : 306.1110

Synthesis of 3-octyl-5'-(tributylstannyl)-[2,2'-bithiophene]-5-carbaldehyde (3)

2.5 M solution of n-butyllithium (n-BuLi) in hexane (7.5 ml, 17.86 mmol) was added dropwise to stirring solution of compound 1-methylpiperazine (1.50 g, 17.86 mmol) in dried THF (25 ml) at -78°C under N_2 atmosphere. After the solution was kept at 78°C for 15 min, compound **2** (2.39 g, 17.86mmol) solution in dried THF (30 ml) was added slowly to the solution and then the solution in flask was allowed to stir for another 20 min. at -78 ^{o}C. Then, 2.5M n-BuLi in hexane was dropped at -78 ^{o}C and the temperature was raised between -70 ^{o}C and -60 ^{o}C and maintained for 1 h. After cooling to ambient, water was added and the mixture was extracted with dichloromethane and washed with water, dried with $MgSO_4$ and concentrated. The crude product was obtained without purification. (2.5 g)

Synthesis of 3-octylthiophene (4).

3-Bromothiophene (8.30 g, 50.93 mmol) was slowly added to a solution of Pd(dppf)Cl_2 (0.37 g, 0.51 mmol) in 40 mL of freshly distilled ethyl ether in a 100 mL flask under nitrogen flux. To the reaction mixture was then dropwise added 33.10 mL of a 2 M octylmagnesium bromide ether solution (66.21 mmol), at a temperature maintained between 0 °C and 5 °C. After addition, the brown solution was stirred at reflux for 15 min. Upon cooling to room temperature, water was carefully added to the reaction, and the resulting mixture was extracted three times with 20 mL of diethyl ether. The combined organic layer was washed with brine, and the organic layer was dried over $MgSO_4$. After filtration, the solvent was removed by rotary evaporation to afford a pale yellow oil, that, on further filtration on celite, gave 5.891 g of the pure compound as a colourless oil (8 g, 80%). ^{1}H NMR (300 MHz, CD_2Cl_2, δ): 7.27-7.30 (q, 1H), 7.49-7.51 (q, 1H), 6.97-7.00 (m, 2H), 2.65-2.70 (t, 2H), 1.62-1.72 (m, 2H), 1.33-1.38 (m, 10H), 0.92-0.96(m, 3H)

Synthesis of diethyl 2,5-bis(4-octylthiophen-2-yl)terephthalate (5).

2.5 M solution of n-butyllithium (n-BuLi) in hexane (12.22 mL) was added dropwise to stirring solution of compound **4** (5.71 g, 29.09 mmol) in dried THF (60 ml) at -78°C under N_2. After dropping 2.5M n-BuLi, the solution was slowly raised to -35°C. Dried zinc chloride (3.96 g, 29.09 mmol) solution in dried THF (40 mL) was added slowly to the solution and then the solution in flask was allowed to stir for 1 h at -35°C. Diethyl 2,5-dibromoterephthalate (4.98 g, 13.09 mmol) and Pd(PPh_3)$_4$ (0.71 g, 0.61 mmol) were added into the solution of 4-octylthiophen-2-ylzinc(II) chloride under N_2, and then the mixture was refluxed for 8 h. After cooling to ambient, water was added and the mixture was extracted with dichloromethane and washed with water, dried with $MgSO_4$ and concentrated. The residues was purified by column chromatography using methylene chloride:n-hexane (v:v=1:1) as eluent to get the product as an light green oil (4 g, 50%). ^{1}H NMR (300 MHz, CD_2Cl_2, δ): 7.79 (s, 1H), 7.04-7.05 (d, J = 1.36 Hz, 2H), 6.99-7.00 (d, J = 1.36 Hz, 2H), 4.23-4.30 (q, 4H), 2.64-2.69 (t, 4H), 1.63-1.73 (m, 4H), 1.34-1.38 (m, 20H), 1.20-1.25 (t, 6H), 0.91-0.96 (t, 6H). HR-MS (FAB) m/z $C_{36}H_{50}O_4S_2$ Cacld : 610.3151, Found : 610.3141

H-NMR, 13C-NMR spectra and Mass spectra of compounds

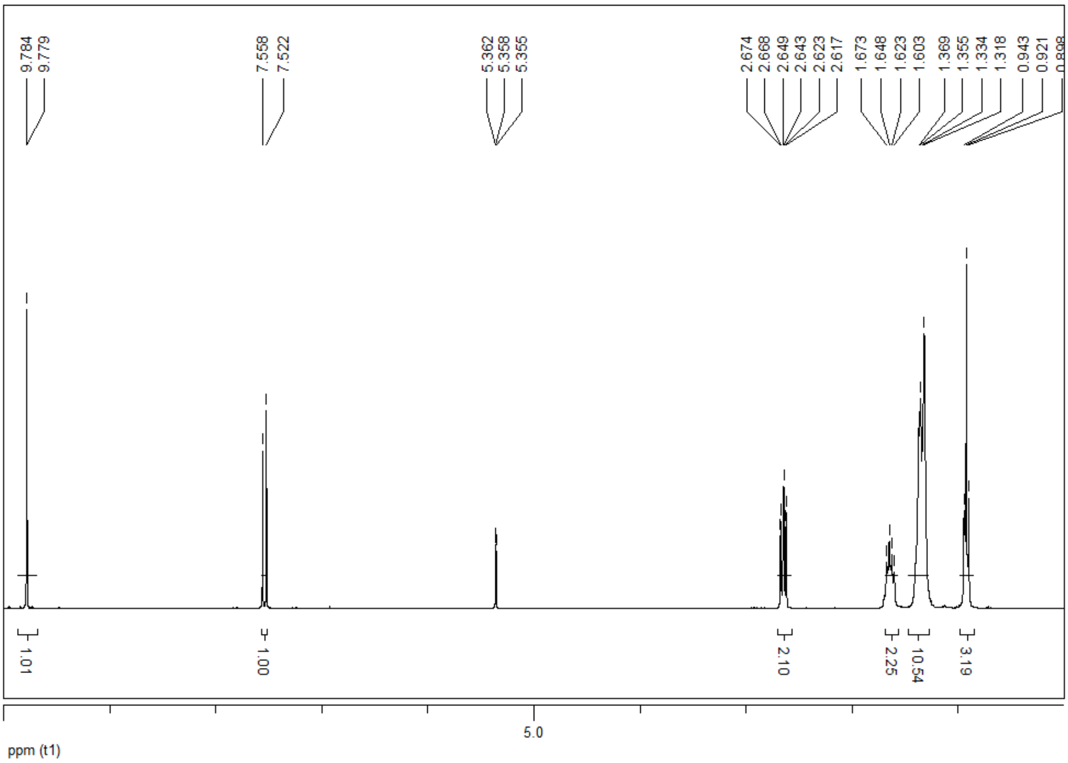

Figure S1. ¹H NMR data of 5-bromo-4-octylthiophene-2-carbaldehyde (1)

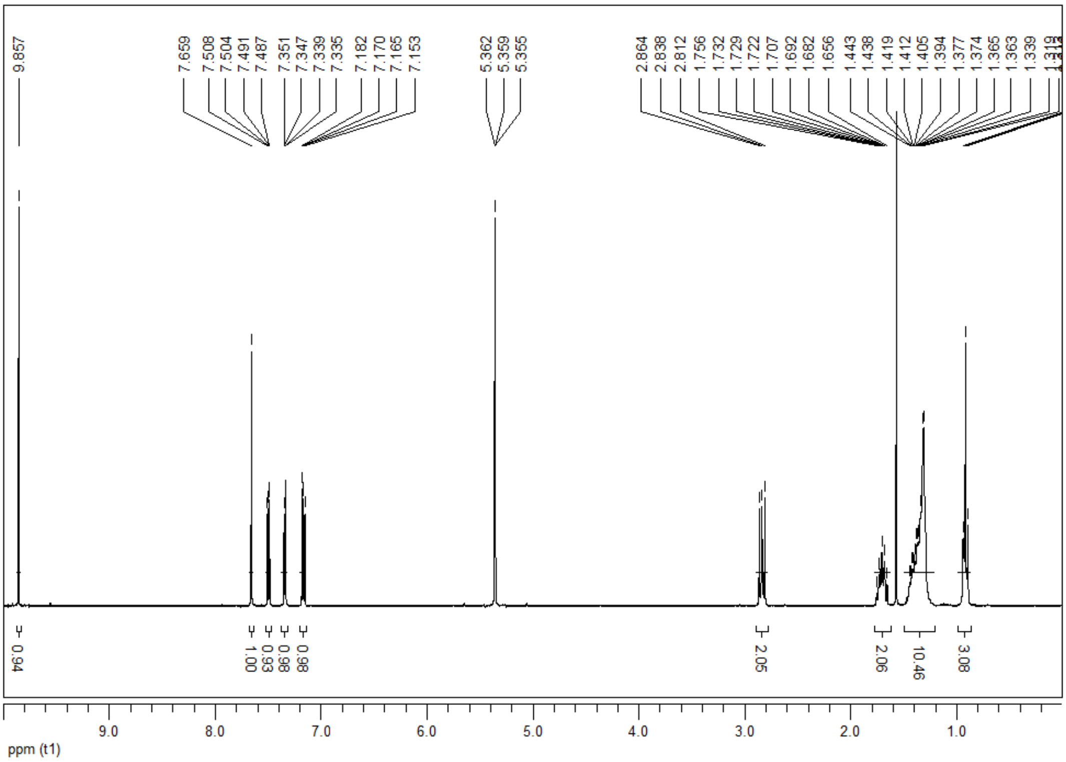

Figure S2. ¹H NMR data of 3-octyl-[2,2'-bithiophene]-5-carbaldehyde (2)

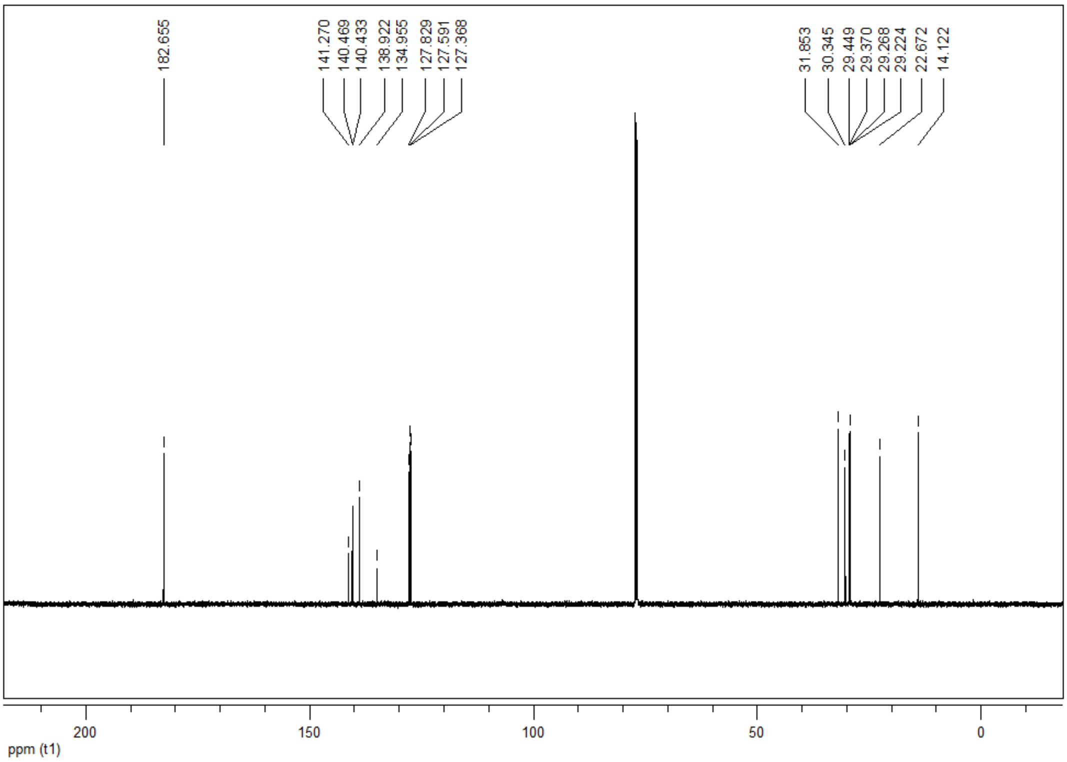

Figure S3. ^{13}C NMR data of 3-octyl-[2,2'-bithiophene]-5-carbaldehyde (2)

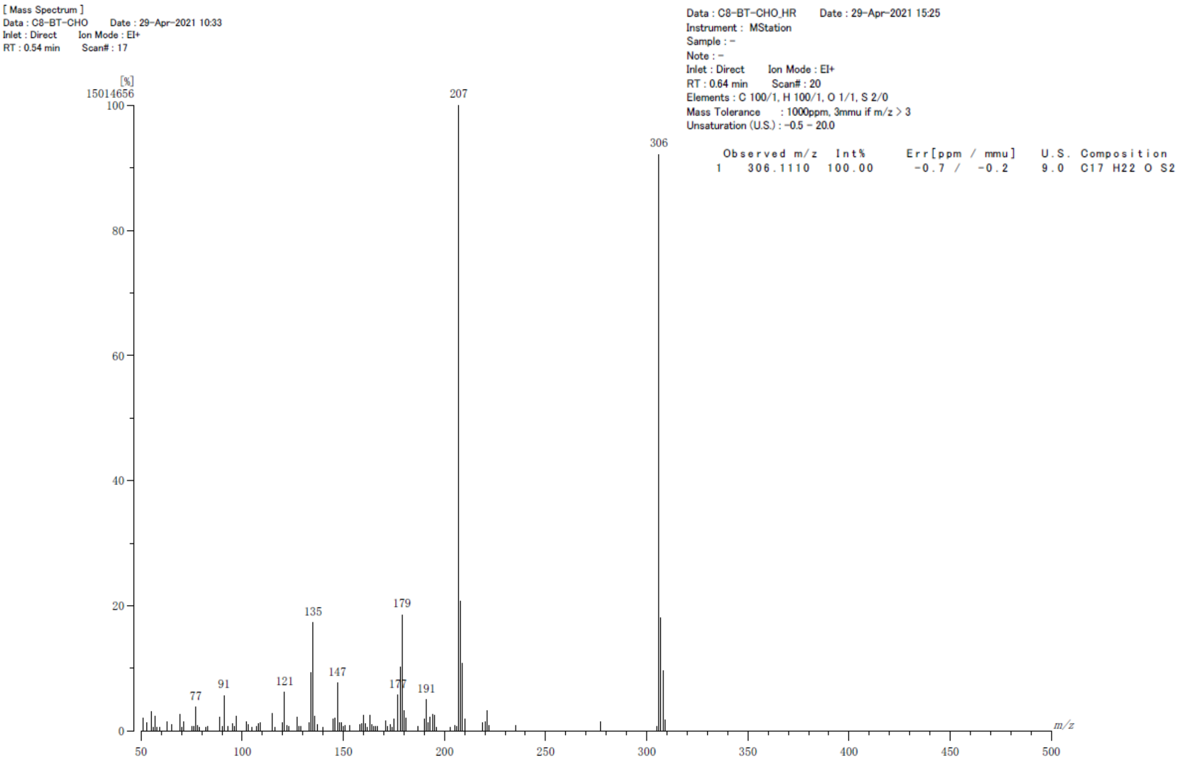

Figure S4. EI Mass data of 3-octyl-[2,2'-bithiophene]-5-carbaldehyde (2)

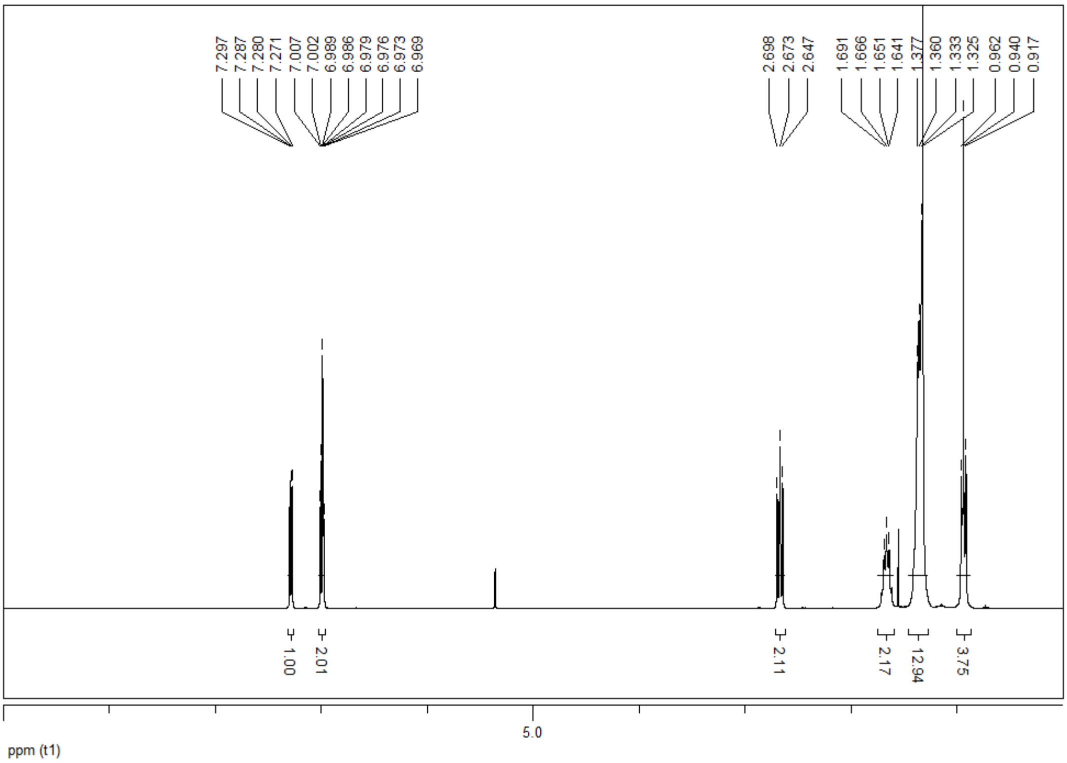

Figure S5. ^{1}H NMR data of 3-octylthiophene (4)

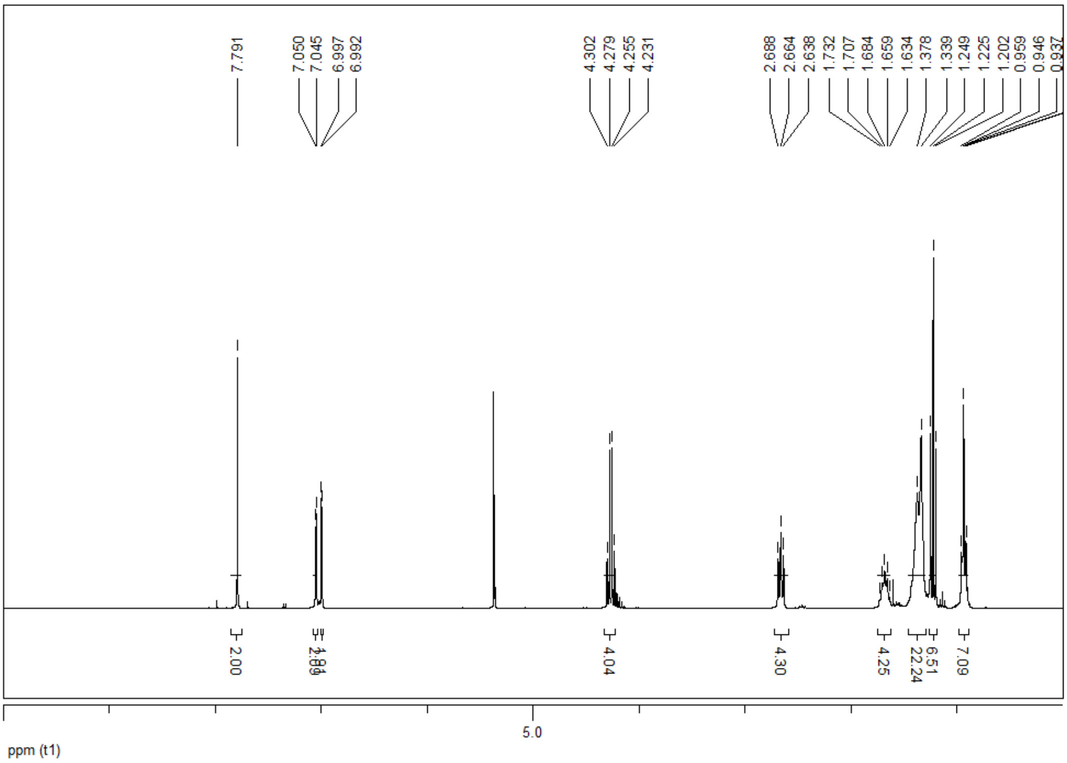

Figure S6. ^{1}H NMR data of diethyl 2,5-bis(4-octylthiophen-2-yl)terephthalate (5)

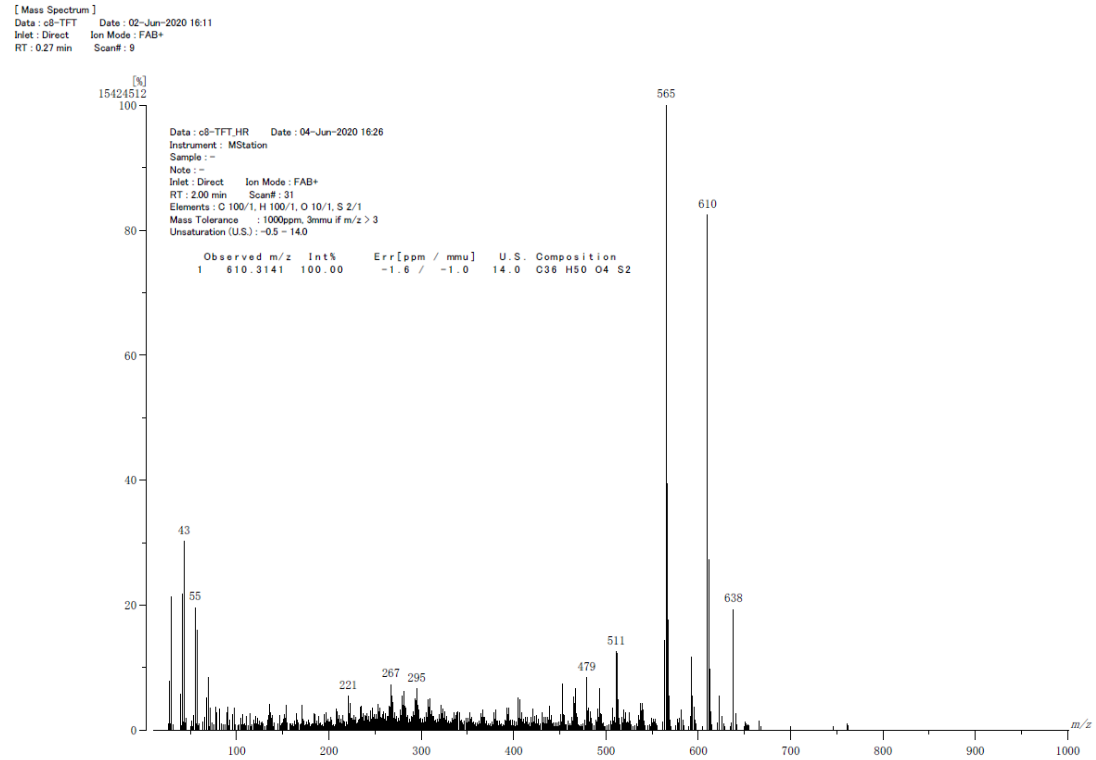

Figure S7. FAB Mass data of diethyl 2,5-bis(4-octylthiophen-2-yl)terephthalate (5)

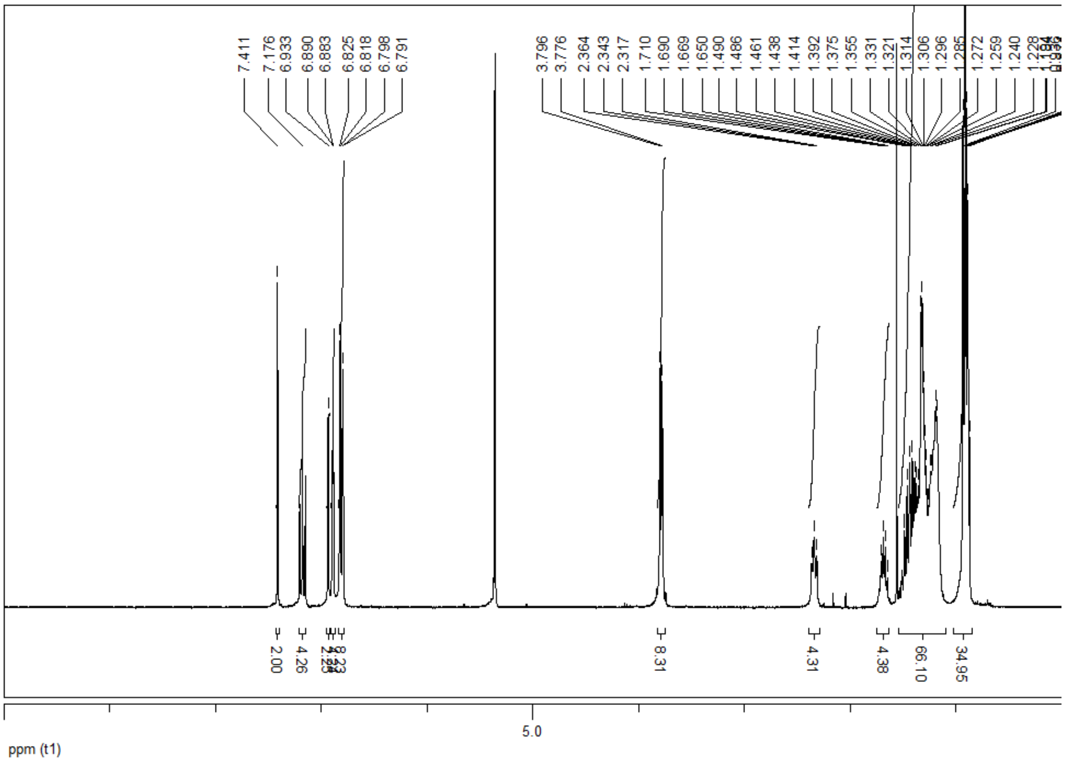

Figure S8. ^{1}H NMR data of 4,4,9,9-tetrakis(3-((2-ethylhexyl)oxy)phenyl)-3,8-dioctyl-4,9-dihydro-s-indaceno[1,2-b:5,6-b']dithiophene (6)

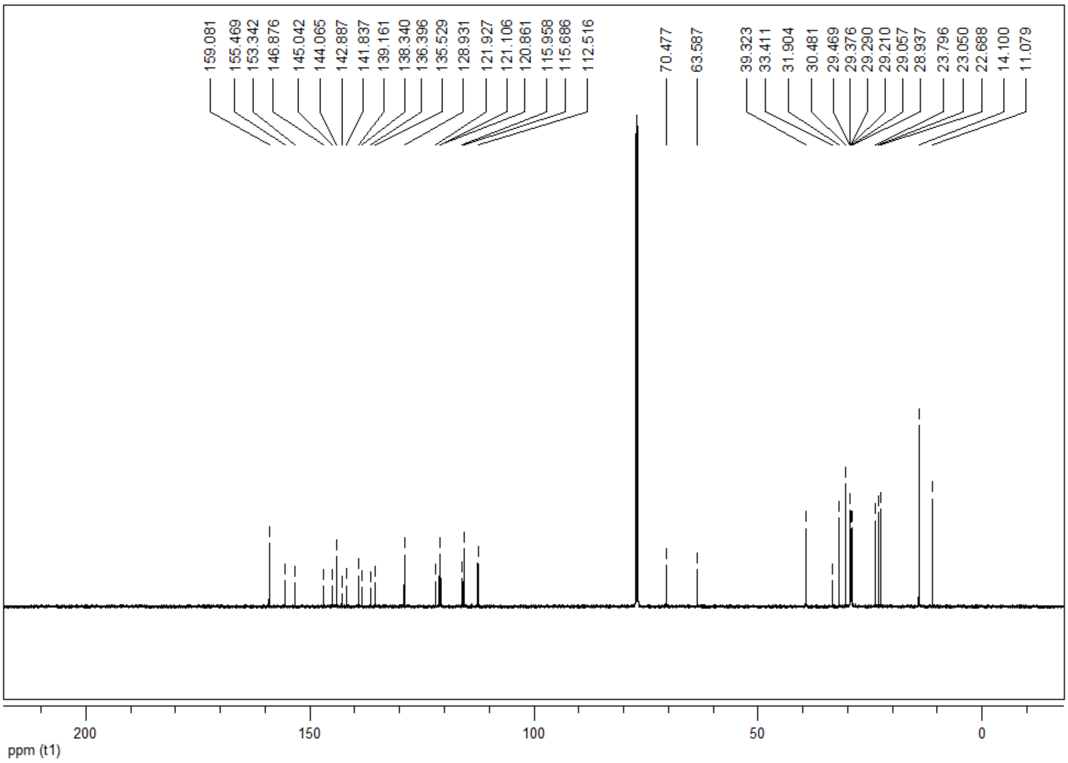

Figure S9. ^{13}C NMR data of 4,4,9,9-tetrakis(3-((2-ethylhexyl)oxy)phenyl)-3,8-dioctyl-4,9-dihydro-s-indaceno[1,2-b:5,6-b']dithiophene (6)

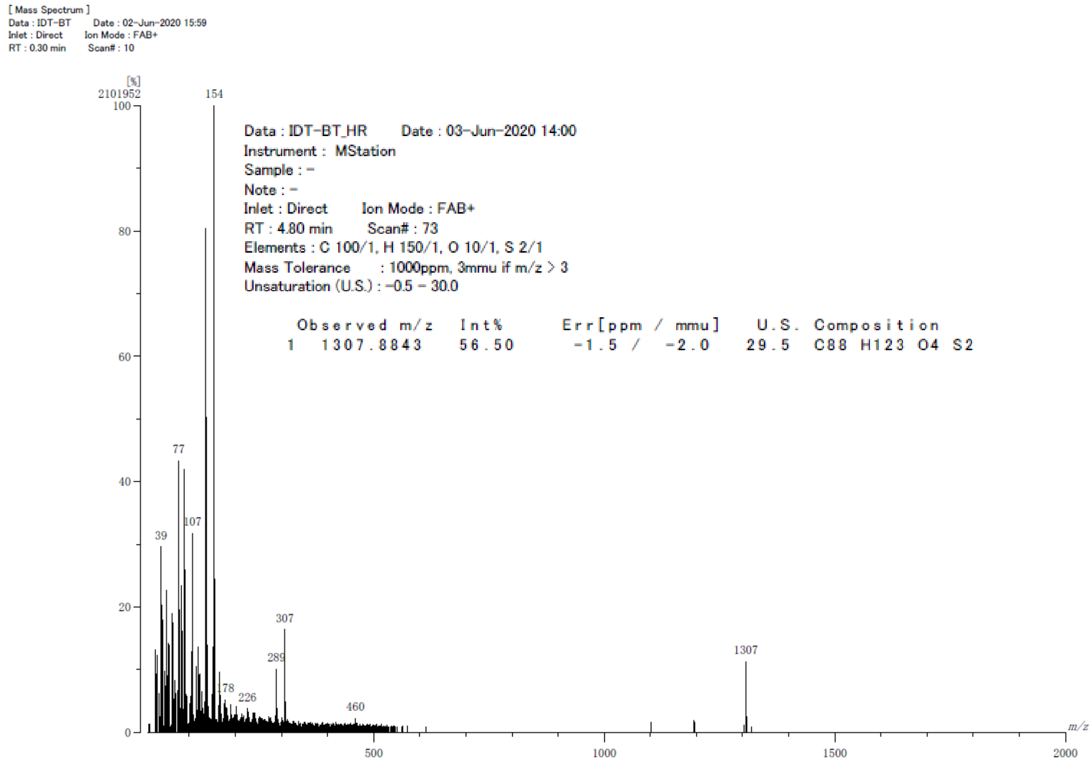

Figure S10. FAB Mass data of 4,4,9,9-tetrakis(3-((2-ethylhexyl)oxy)phenyl)-3,8-dioctyl-4,9-dihydro-s-indaceno[1,2-b:5,6-b']dithiophene (6)

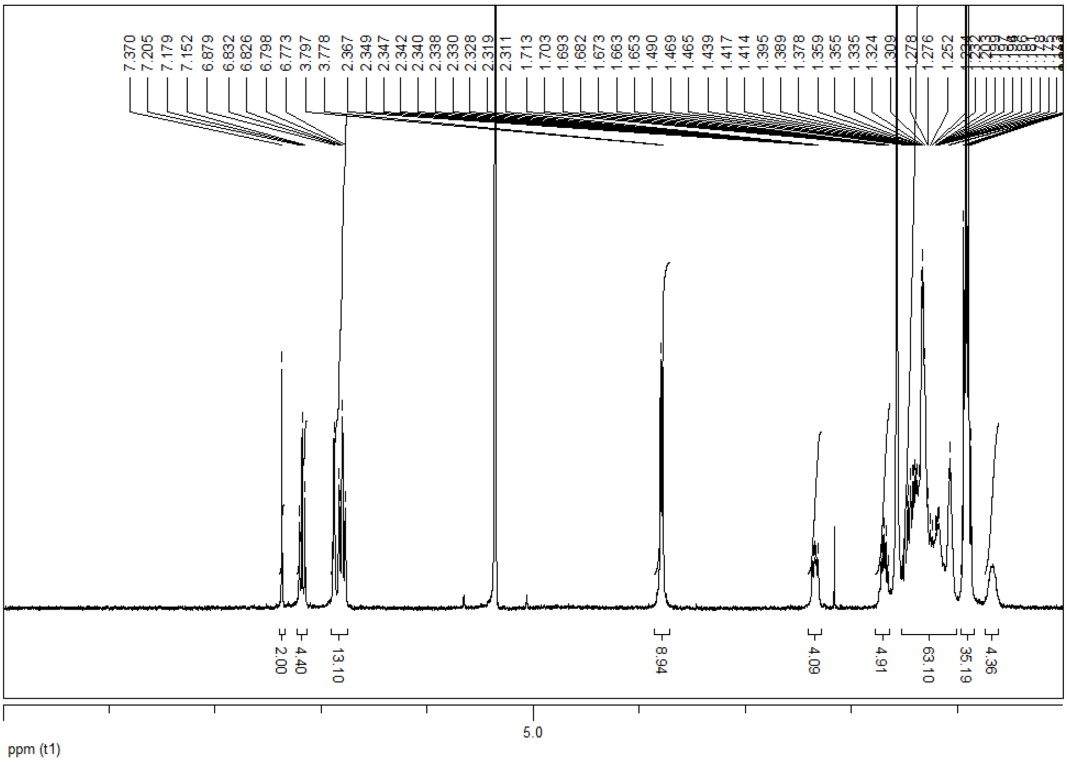

Figure S11. ^{1}H NMR data of 2,7-dibromo-4,4,9,9-tetrakis(3-((2-ethylhexyl)oxy)phenyl)-3,8-dioctyl-4,9-dihydro-s-indaceno[1,2-b:5,6-b']dithiophene (7)

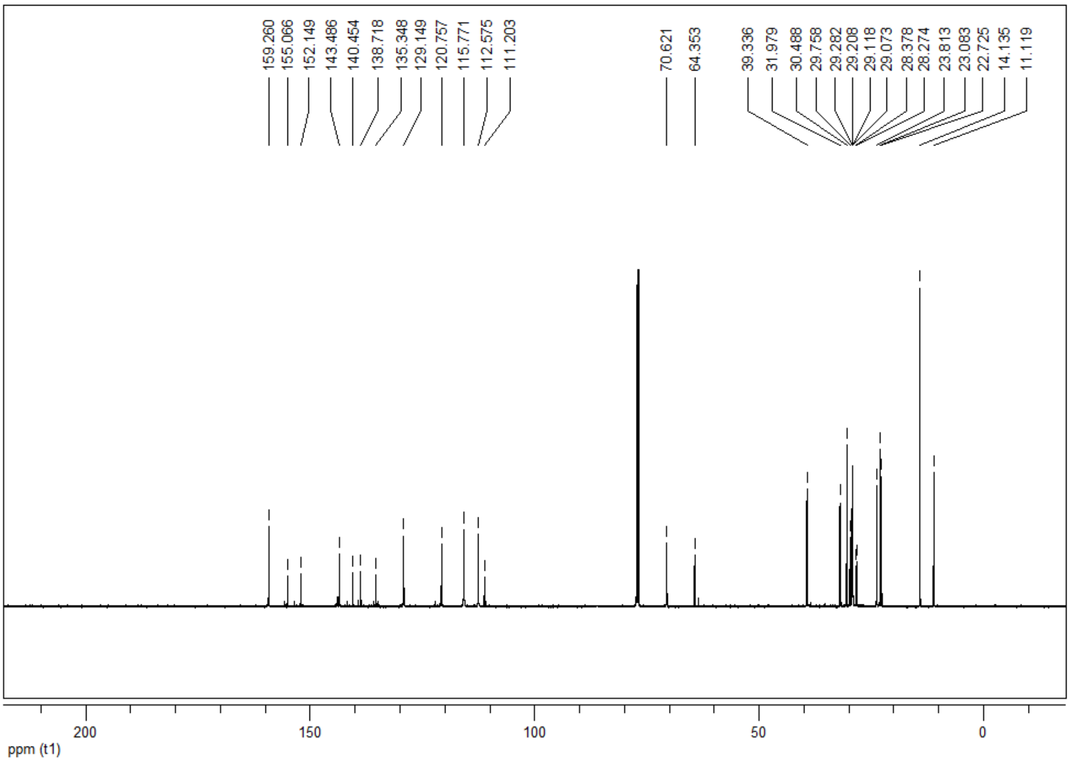

Figure S12. ^{13}C NMR data of 2,7-dibromo-4,4,9,9-tetrakis(3-((2-ethylhexyl)oxy)phenyl)-3,8-dioctyl-4,9-dihydro-s-indaceno[1,2-b:5,6-b']dithiophene (7)

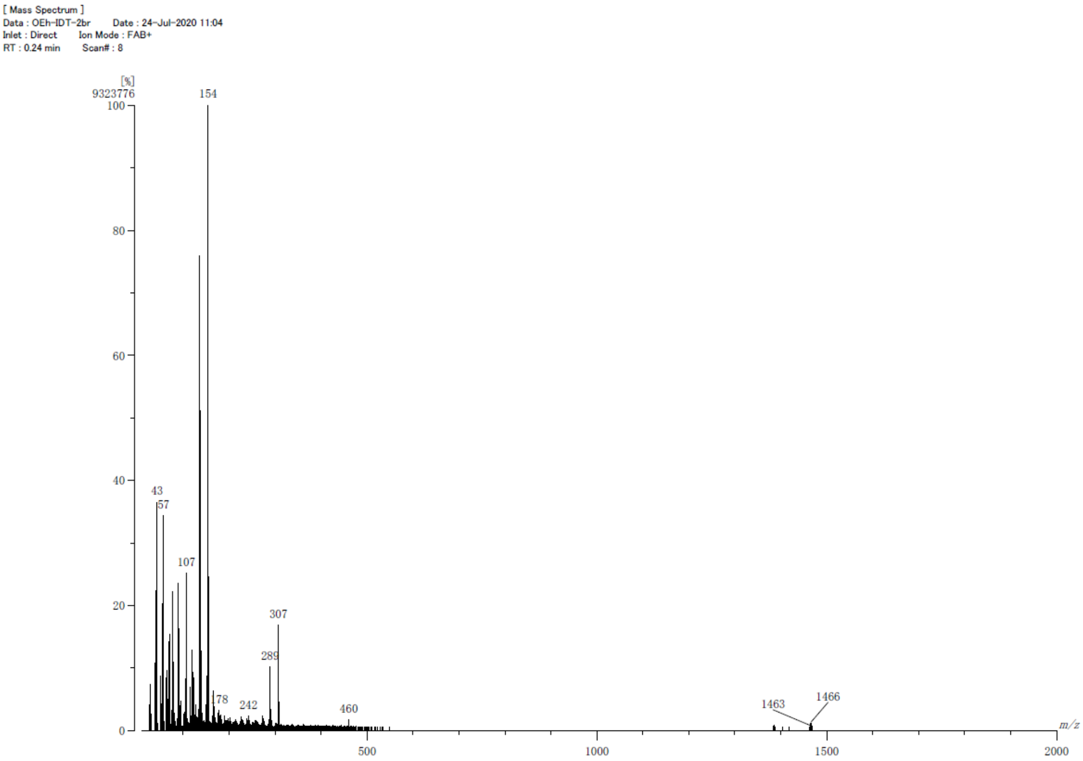

Figure S13. FAB Mass data of 2,7-dibromo-4,4,9,9-tetrakis(3-((2-ethylhexyl)oxy)phenyl)-3,8-dioctyl-4,9-dihydro-s-indaceno[1,2-b:5,6-b']dithiophene (7)

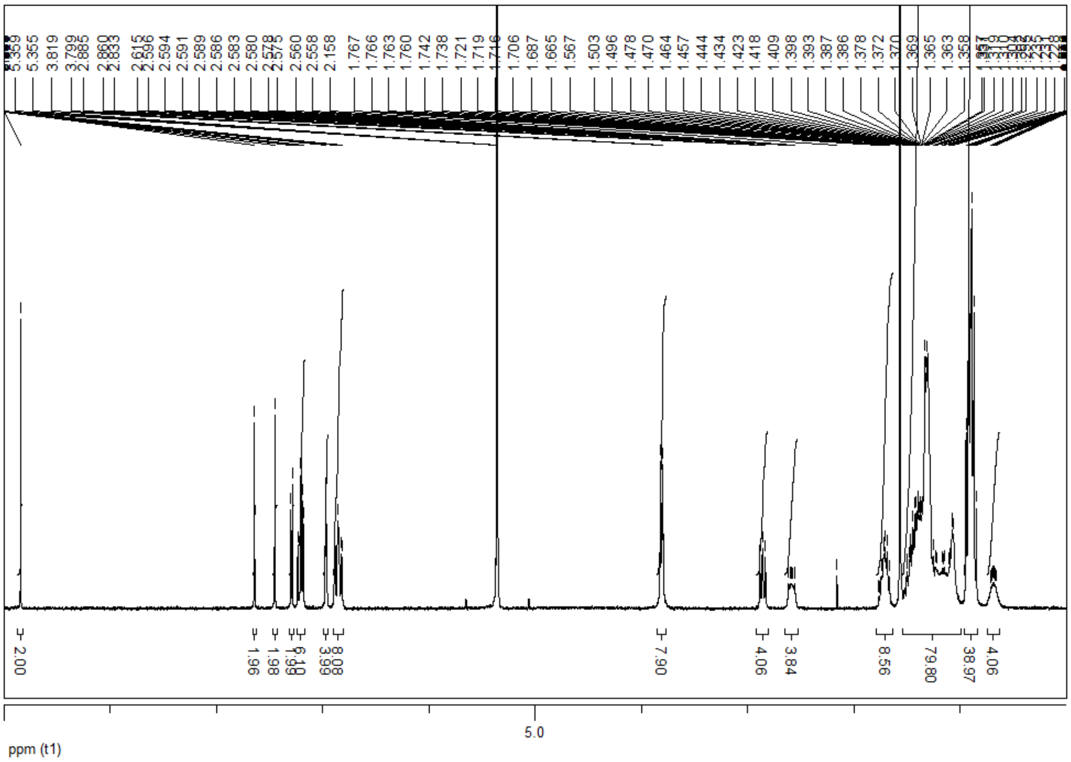

Figure S14. ¹H NMR data of 5',5'''-(4,4,9,9-tetrakis(3-((2-ethylhexyl)oxy)phenyl)-3,8-dioctyl-4,9-dihydro-s-indaceno[1,2-b:5,6-b']dithiophene-2,7-diyl)bis(3-octyl-[2,2'-bithiophene]-5-carbaldehyde) (8)

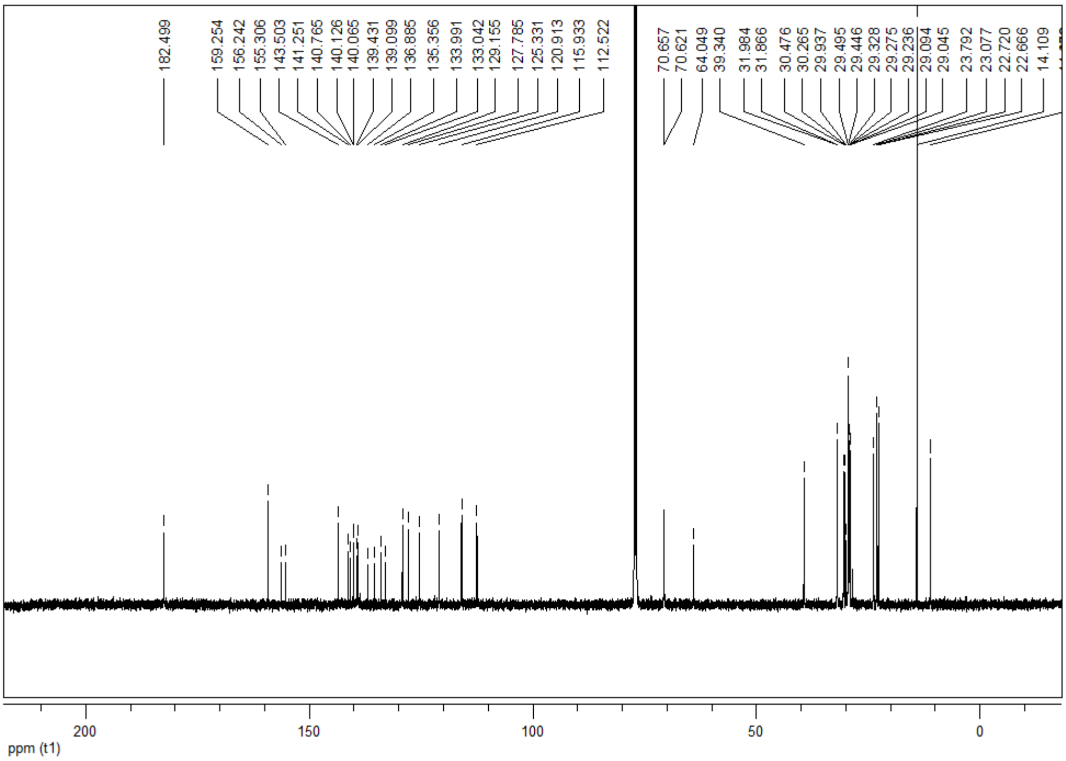

Figure S15. ^{13}C NMR data of 5',5'''-(4,4,9,9-tetrakis(3-((2-ethylhexyl)oxy)phenyl)-3,8-dioctyl-4,9-dihydro-s-indaceno[1,2-b:5,6-b']dithiophene-2,7-diyl)bis(3-octyl-[2,2'-bithiophene]-5-carbaldehyde) (8)

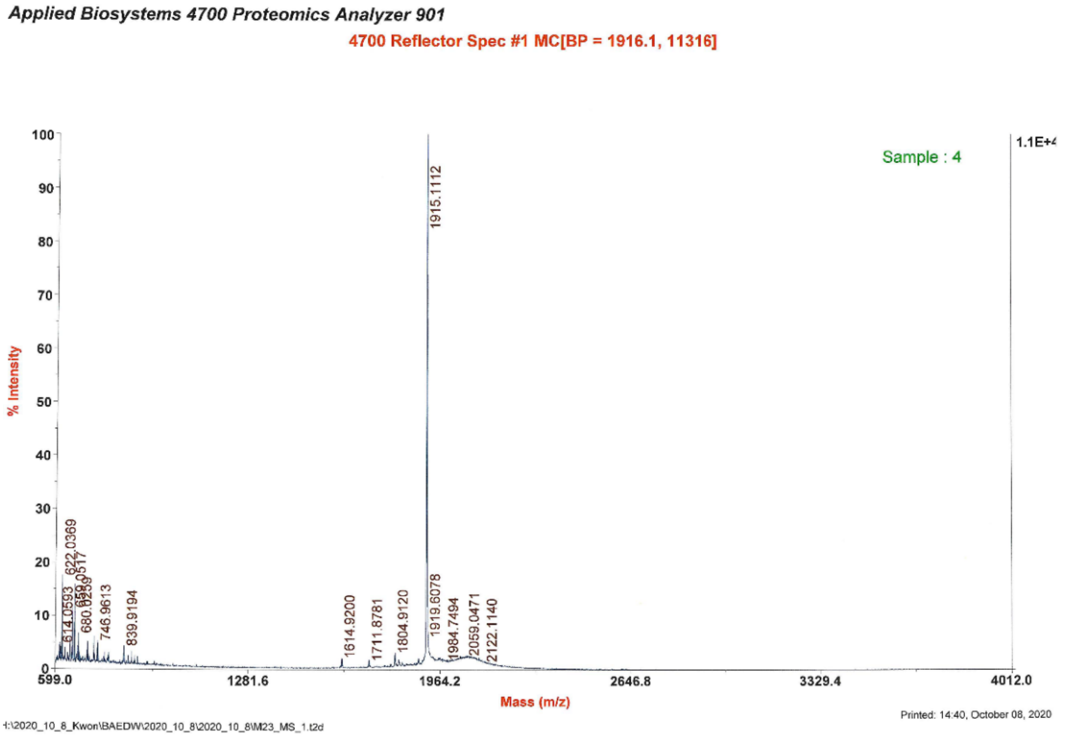

Figure S16. MALDI-TOF Mass data of 5',5'''-(4,4,9,9-tetrakis(3-((2-ethylhexyl)oxy)phenyl)-3,8-dioctyl-4,9-dihydro-s-indaceno[1,2-b:5,6-b']dithiophene-2,7-diyl)bis(3-octyl-[2,2'-bithiophene]-5-carbaldehyde) (8)

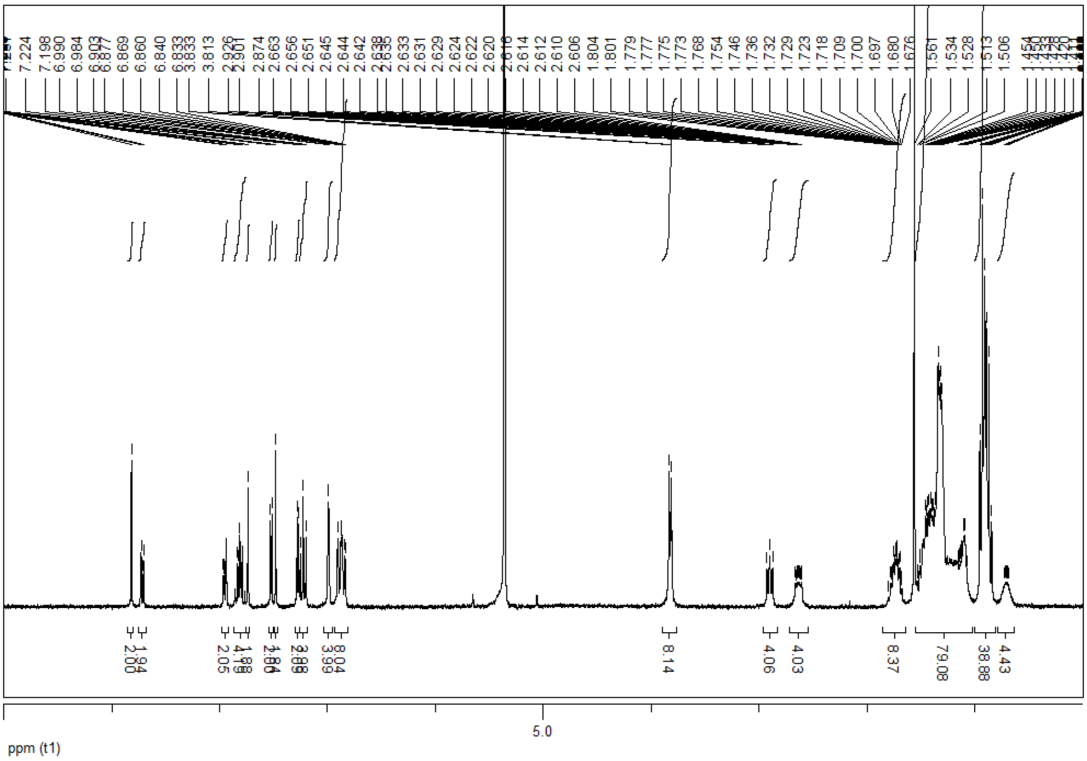

Figure S17. ^{1}H NMR data of IDT-BT-IC

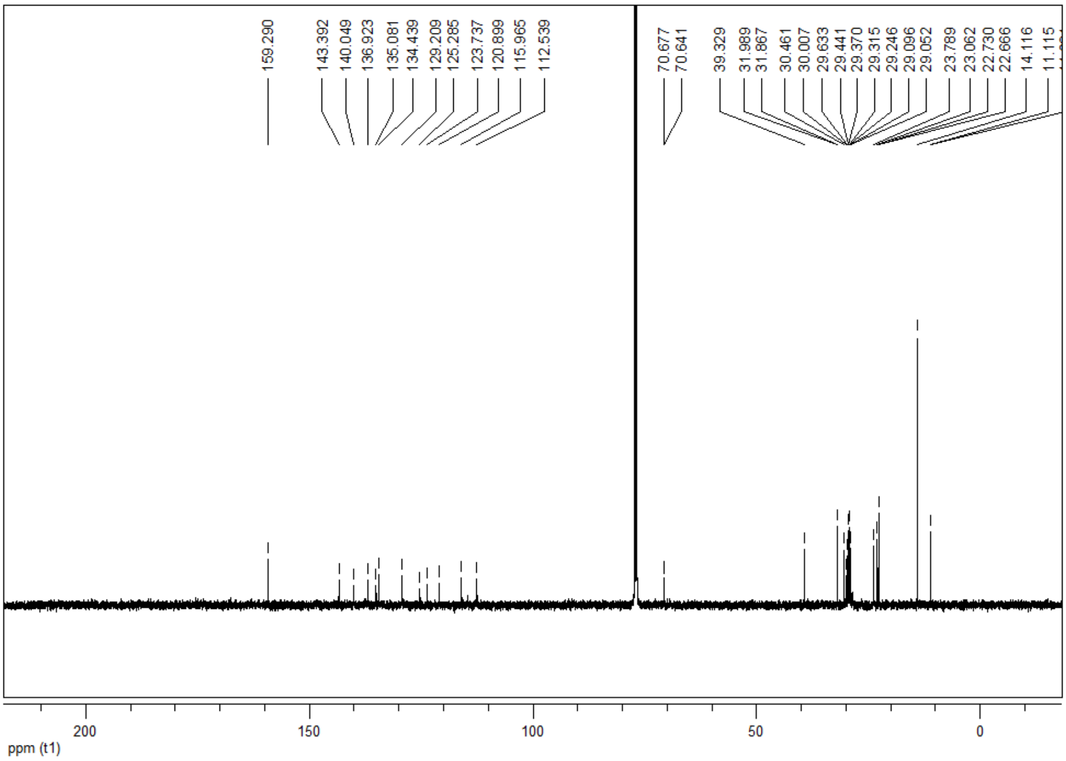

Figure S18. ^{13}C NMR data of IDT-BT-IC

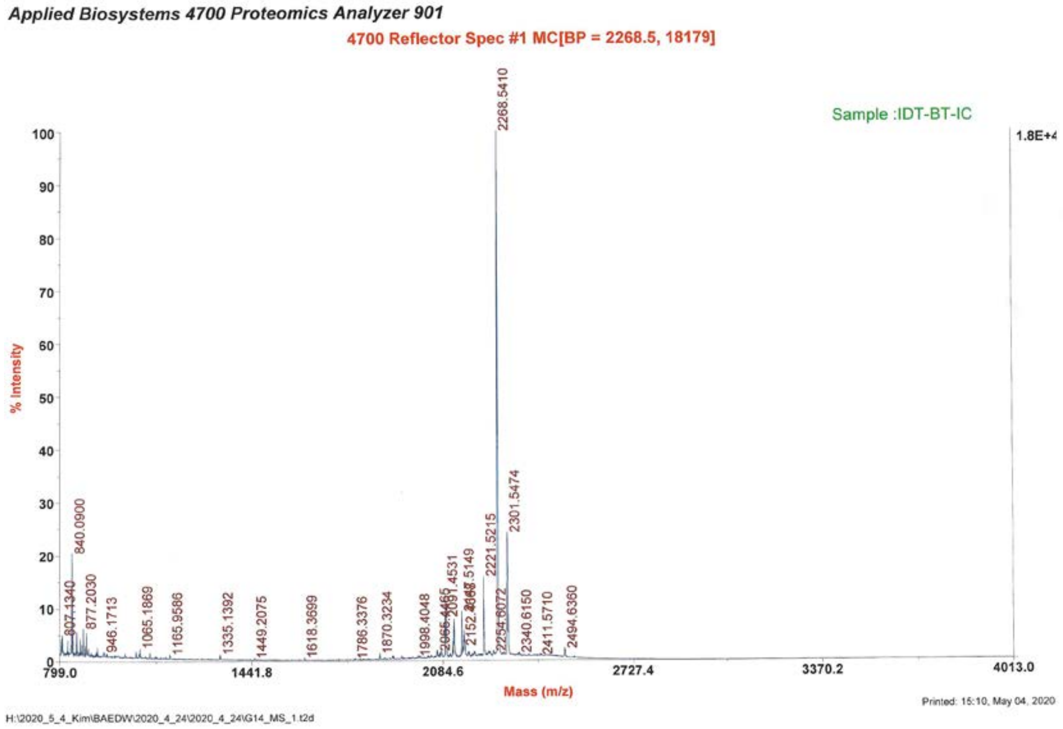

Figure S19. MALDI-TOF Mass data of IDT-BT-IC

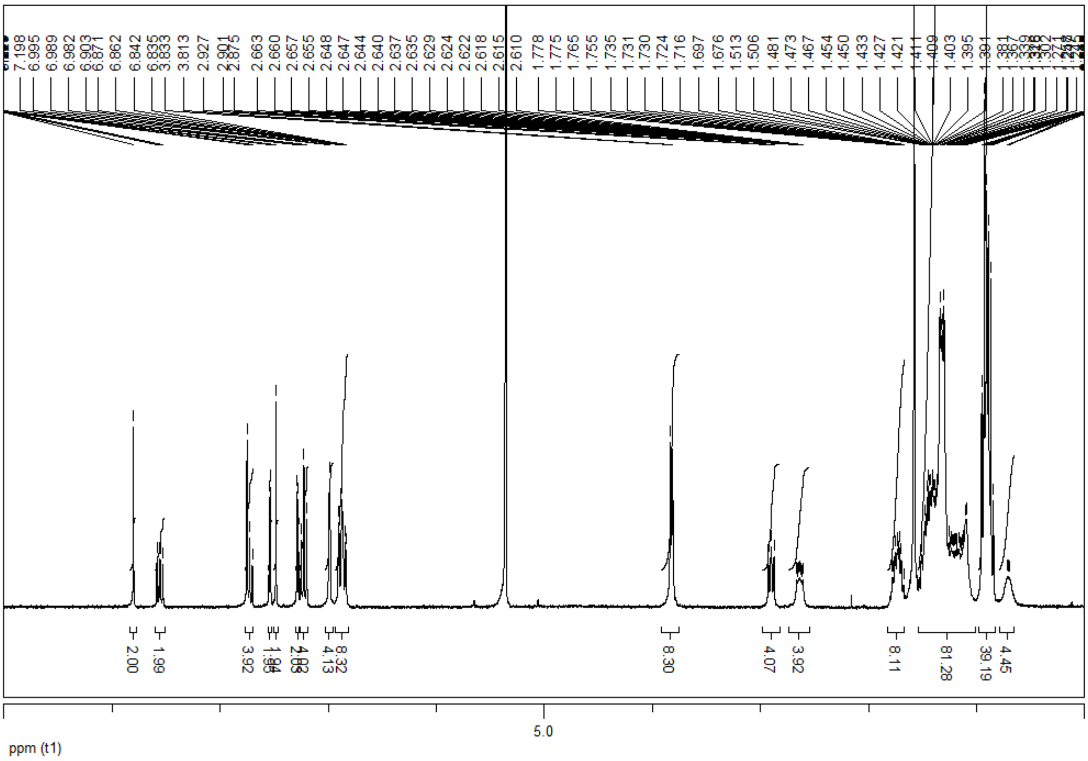

Figure S20. ^{1}H NMR data of IDT-BT-IC4F

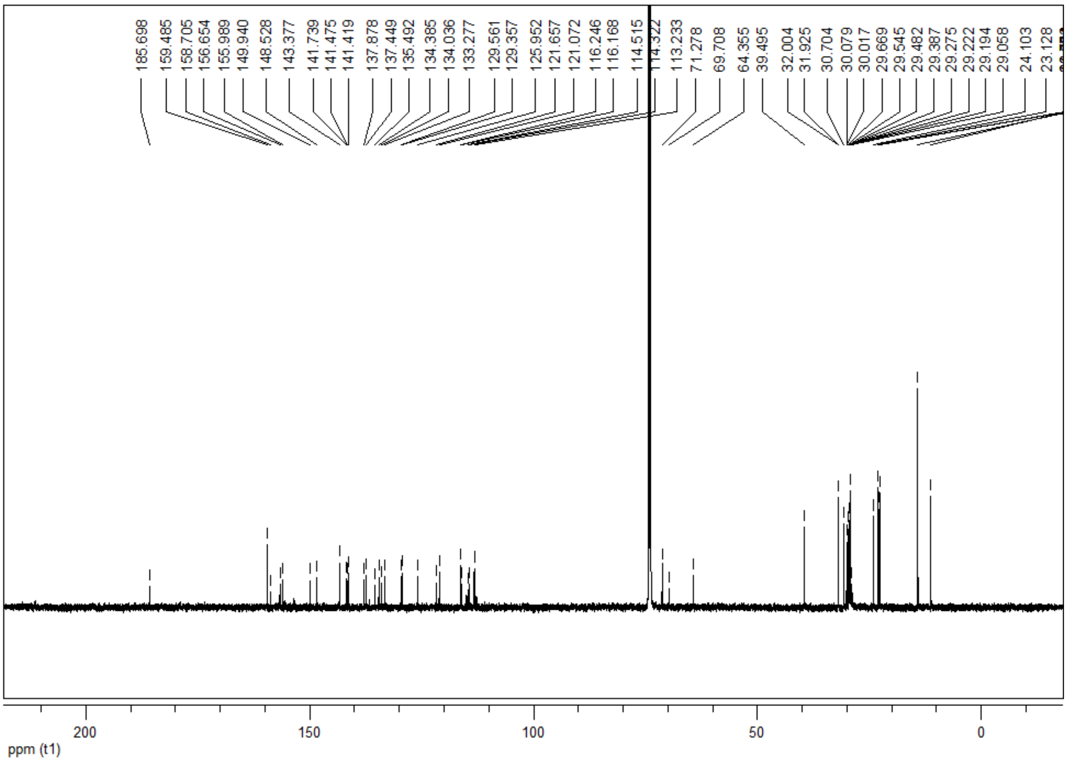

Figure S21. ^{13}C NMR data of IDT-BT-IC4F

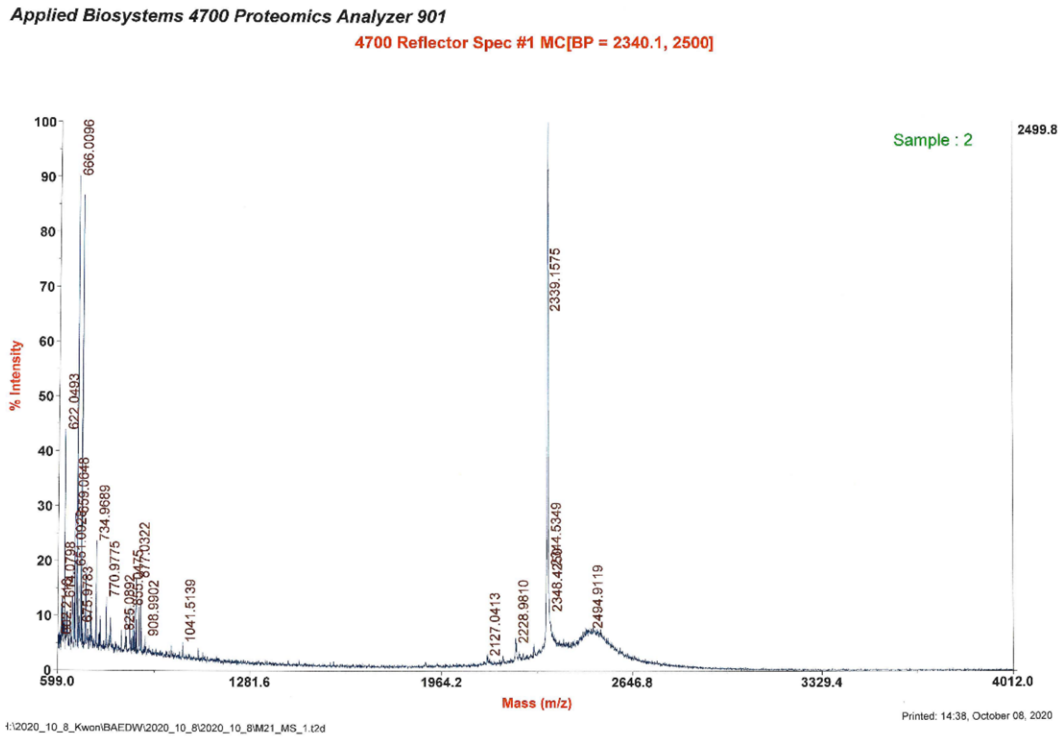

Figure S22. MALDI-TOF Mass data of IDT-BT-IC4F

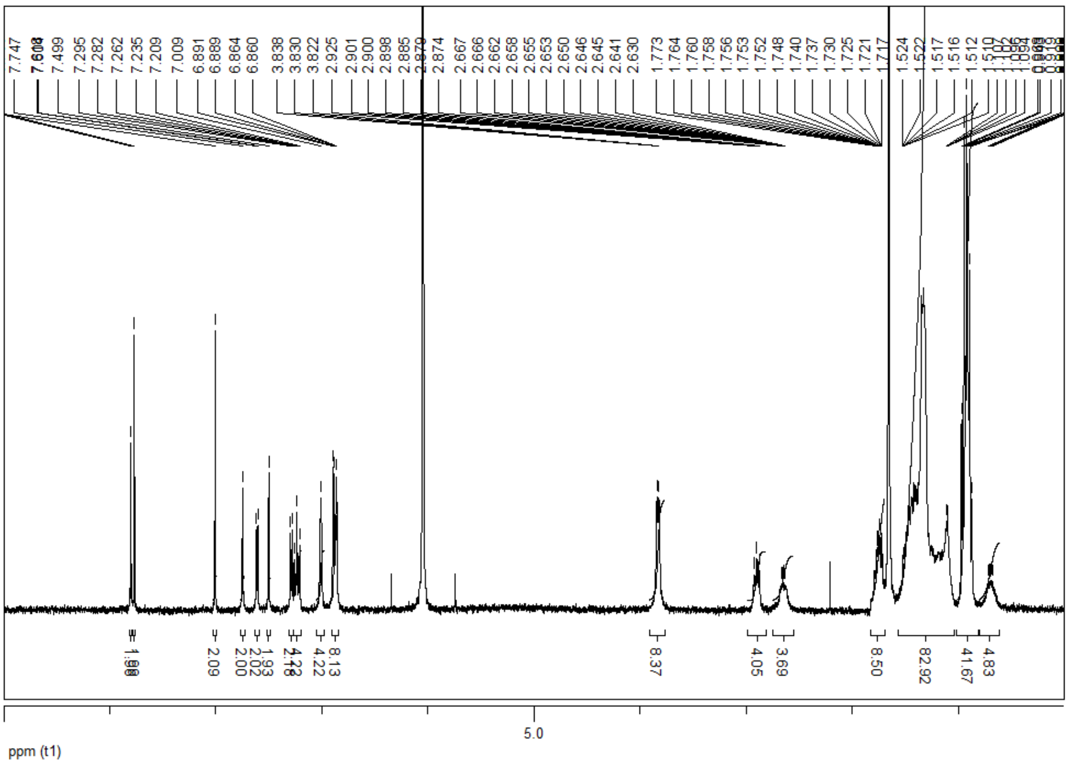

Figure S23. ^{1}H NMR data of IDT-BT-IC4Cl

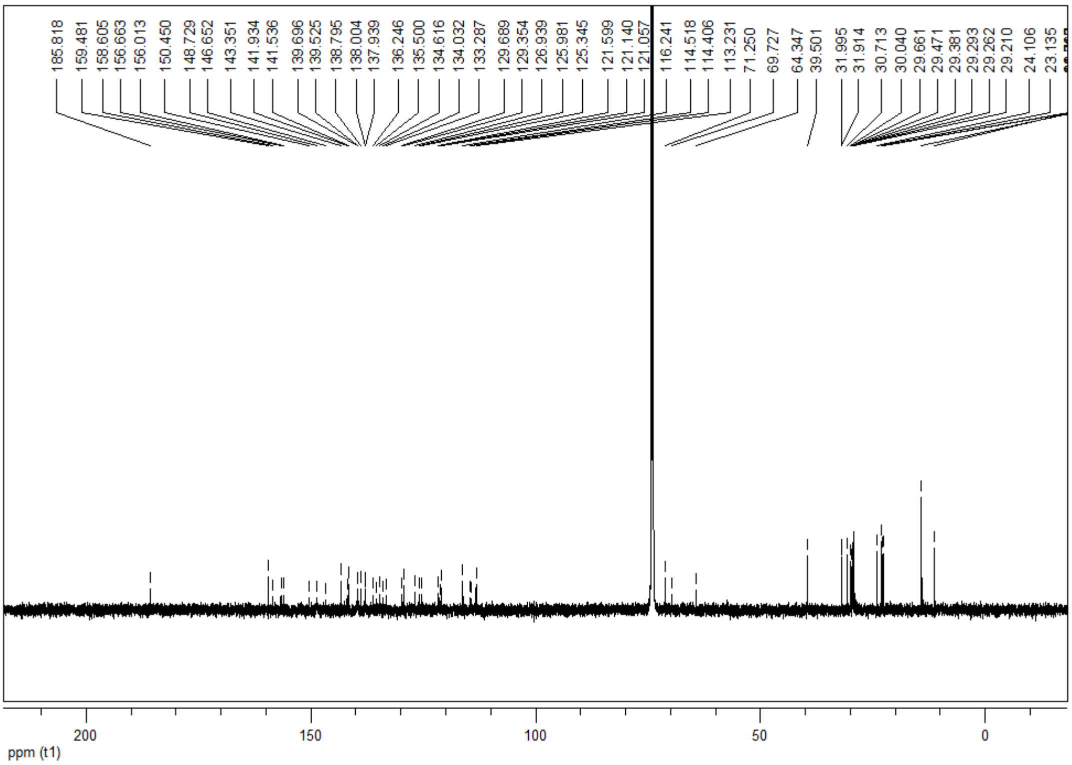

Figure S24. ^{13}C NMR data of IDT-BT-IC4Cl

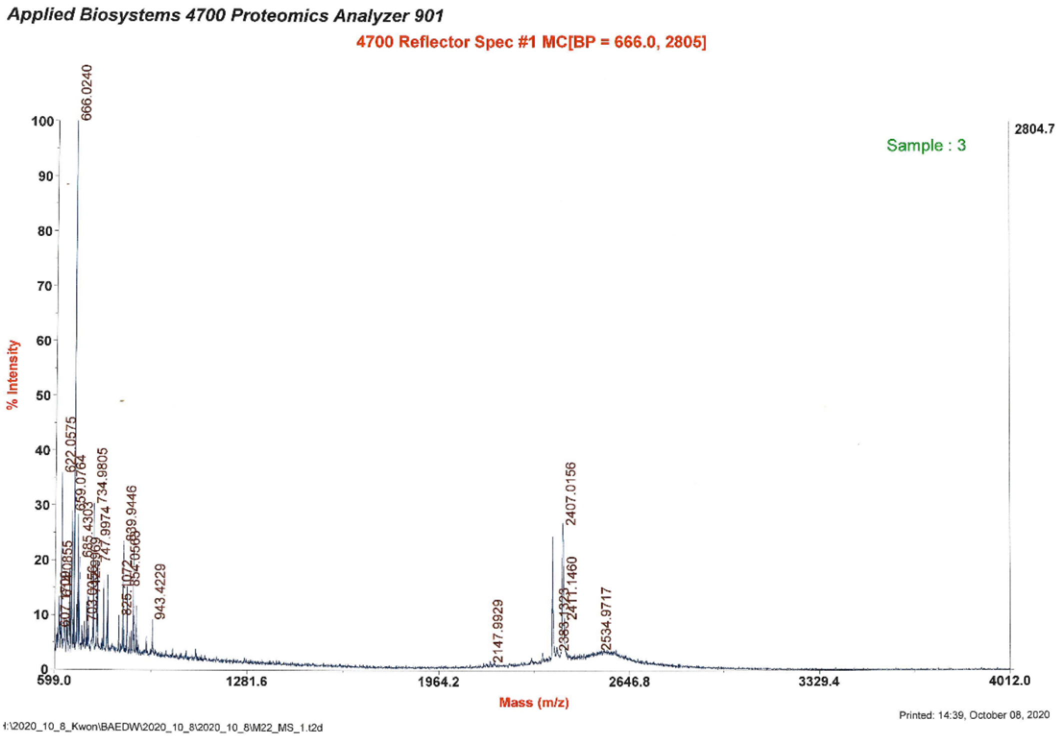

Figure S25. MALDI-TOF Mass data of IDT-BT-IC4Cl

Normalized absorption UV-Vis spectra of compounds

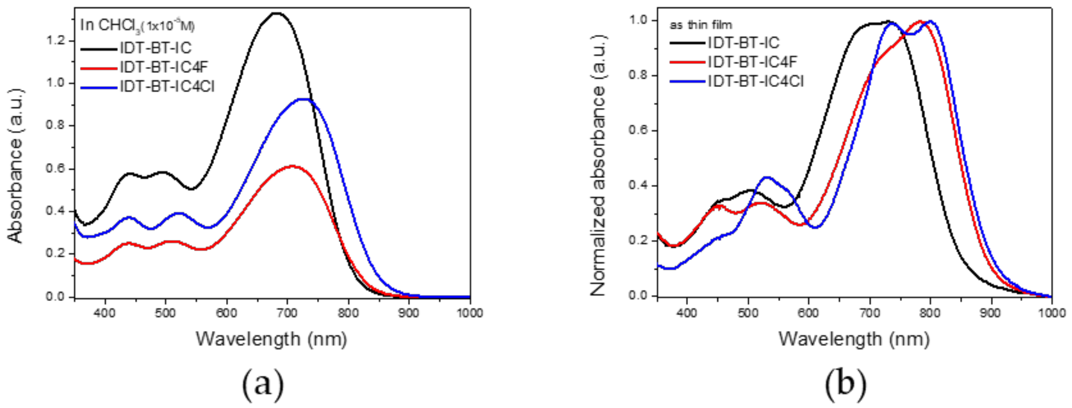

Figure S26. UV-Vis absorption (a) in $CHCl_3$, (b) as thin film of IDT-BT-IC, IDT-BT-IC4F and IDT-BT-IC4Cl

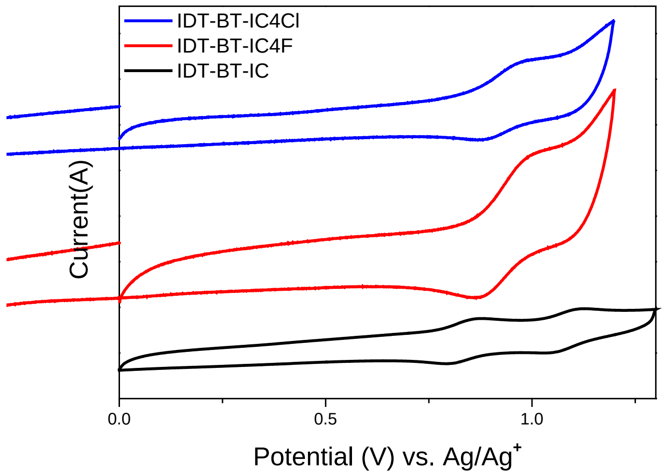

Figure S27. Cyclic voltammetry of IDT-BT-IC, IDT-BT-IC4F and IDT-BT-IC4Cl

Thermal properties

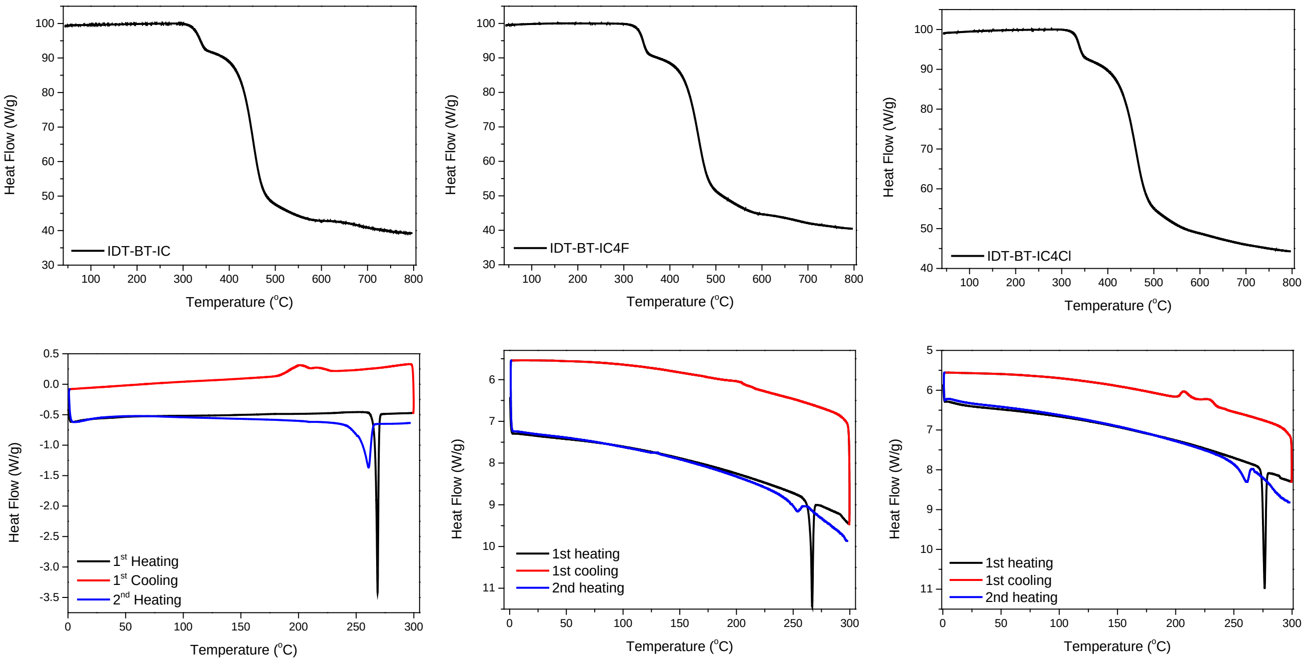

Figure S28. TGA curve and DSC thermograms of NFAs

Table S1. Summaries of optical and electrochemical properties of NFAs

	λ_{max}^{S} (nm)	λ^{f} (nm)	UV-edge (nm)	Molar extinction coefficient ($cm^{-1}\cdot M^{-1}$)	E_g (eV)	HOMO (eV)	LUMO (eV)
IDT-BT-IC	682	730	869	132800	1.43	-5.25	-3.82
IDT-BT-IC4F	710	785	890	61100	1.39	-5.25	-3.86
IDT-BT-IC4Cl	726	736, 799	896	92700	1.38	-5.26	-3.88

Table S2. Summaries of thermal properties of NFAs

	$T_{d,}$$^{0.01\%}$ (°C)	$T_{d,}$$^{5\%}$ (°C)	T_g (°C)	T_m (°C)	T_c (°C)
IDT-BT-IC	297	338	-	269	208
IDT-BT-IC4F	294	341	130	267	204
IDT-BT-IC4Cl	304	340	-	276	229
